# ﻿Comprehensive genome analysis of two *Cytospora* (Cytosporaceae, Diaporthales) species associated with canker disease of spruce: *C.piceae* and *C.piceicola* sp. nov.

**DOI:** 10.3897/mycokeys.117.145445

**Published:** 2025-05-05

**Authors:** Evgeny Ilyukhin, Yanpeng Chen, Svetlana Markovskaja, Ashwag Shami, Sajeewa S. N. Maharachchikumbura

**Affiliations:** 1 Saskatchewan S9H, Canada Unaffiliated Saskatchewan Canada; 2 Center for Informational Biology, School of Life Science and Technology, University of Electronic Science and Technology of China, Chengdu 611731, China University of Electronic Science and Technology of China Chengdu China; 3 Laboratory of Mycology, Nature Research Centre, LT 08406 Vilnius, Lithuania Laboratory of Mycology, Nature Research Centre Vilnius Lithuania; 4 Department of Biology, College of Science, Princess Nourah bint Abdulrahman University, Riyadh, 11671, Saudi Arabia Princess Nourah bint Abdulrahman University Riyadh Saudi Arabia

**Keywords:** Carbohydrate-active enzymes, Cytospora canker, effector proteins, Sordariomycetes, virulence genes

## Abstract

Cytospora canker (CC) is among the most important diseases in conifer trees (*Picea* spp., mainly). This disease poses a significant risk factor for forest health, potentially leading to economic losses for wood producers. To provide a genomic basis of the CC pathogenesis, the genomes of two *Cytospora* species associated with the disease were sequenced and further analyzed within a set of Diaporthales species. The first species was identified as *C.piceae*. The second was described as *C.piceicola***sp. nov.** based on morphological characteristics and multi-gene phylogenetic analysis. The novel species is sister to other *Cytospora* species isolated from conifers. Here, we report 39.7 and 43.8 Mb highly contiguous genome assemblies of *C.piceae* EI-19(A) and *C.piceicola* EI-20, respectively, obtained using Illumina sequencing technology. Despite notably different genome sizes, these species share the main genome characteristics, such as predicted gene number (10,862 and 10,742) and assembly completeness (97.6% and 98.1%). A wide range of genes encoding carbohydrate-active enzymes, secondary metabolite biosynthesis clusters, and secreted effectors were found. Multiple experimentally validated virulence genes were also identified in the studied species. The defined arsenals of enzymes and effectors generally relate to the hemibiotrophic lifestyle with a capability to switch to biotrophy. The obtained evidence also supports that *C.piceae* EI-19(A) and *C.piceicola* EI-20 can cause severe canker disease symptoms in *Picea* spp. specifically. It was additionally observed that the strains of *C.piceae* may have different pathogenicity and virulence characteristics based on the analyses of predicted secondary metabolite complements, effectomes, and virulence-related genes. Phylogenomic analysis and timetree estimations indicated that divergence of the studied species may have occurred relatively late, 11-10 million years ago. Compared to other members of Diaporthales, *C.piceae* EI-19(A) and *C.piceicola* EI-20 implied a moderate rate of gene contraction, but the latter experienced significant gene loss that can additionally support host specificity attributed to these species. But uncovered gene contraction events may point out potential lifestyle differentiation and host shift of the studied species. It was revealed that EI-19(A) and *C.piceicola* EI-20 carry distinct secretomes and effectomes among Diaporthales species. This feature can indicate a species lifestyle and pathogenicity potential. These findings highlight potential targets for identification and/or detection of pathogenic *Cytospora* in conifers. The introduced draft genome sequences of *C.piceae* and *C.piceicola* can be employed as tools to understand basic genetics and pathogenicity mechanisms of fungal species causing canker disease in woody plants. The identified pathogenicity and virulence-related genes would serve as potential candidates for host-induced gene silencing aimed at making plant hosts more resistant to pathogenic species. Furthermore, the comparative genomics component of the study will facilitate the functional analysis of the genes of unknown function in all fungal pathogens.

## ﻿Introduction

The ascomycetous genus *Cytospora* (Cytosporaceae, Diaporthales) includes fungal species occurring mainly on woody plants worldwide. *Cytospora* spp. are found to be associated with more than 600 hosts, including both angiosperms and gymnosperms (Farr and Rossman 2024). The species are usually considered as endophytes or weak pathogens with the latent phase of the life cycle in healthy plants. Some species can cause severe disease symptoms in infected trees under stress, eventually leading to disease outbreaks. The pathogenic *Cytospora* species are often isolated from cankered wood or necrotic lesions developing on branches and twigs of affected trees (Zhu et al. 2020; Eken and Sevindik 2024; Lin et al. 2024; Wang et al. 2024).

**Figure 1.**
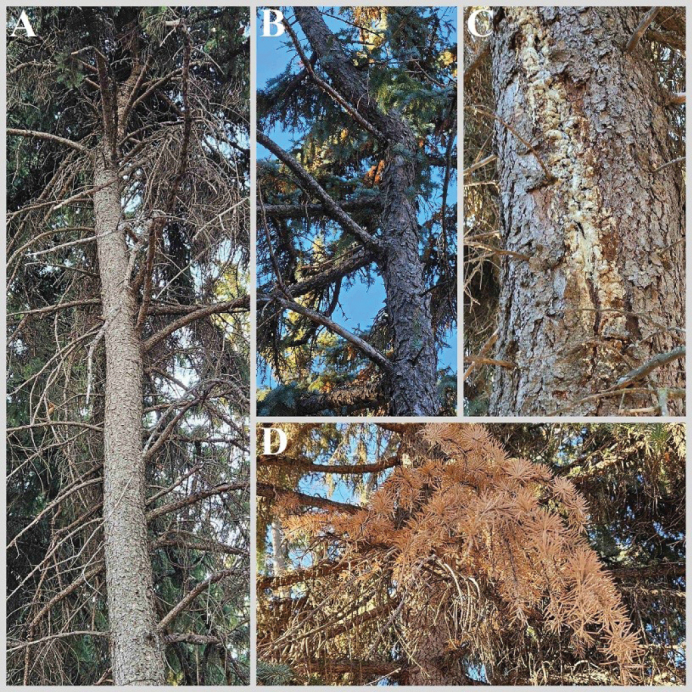
*Picea* spp. with typical CC symptoms: branch dieback (**A**), stem deformation (**B**), resin coating (**C**), and needle yellowing (**D**).

Cytospora canker (CC) is one of the most common diseases of spruce (*Picea* spp.) and fir (*Abies* spp.) in North America. In Canada, forests cover about half of the territory with a predominance of conifers (67.8%) (The State of Canada’s Forests 2023). Thus, wood canker pathogens can cause significant ecological and economic consequences. The canker disease usually progresses when a tree becomes stressed or injured by common abiotic factors such as drought or ice damage (Proffer and Hart 1988). Cankers form on branches and are often coated in a thick layer of resin, causing deformation, growth reduction, dieback, needle yellowing, premature defoliation, and occasionally tree collapse (Pan et al. 2018). The disease decreases wood quality or makes trees hideous when used as ornamental plants (Fig. 1).

Early and accurate species identification is crucial for disease management practices. Previously, Cytospora (Valsa) kunzei was the only species reported as a causal agent of CC in the region (Kamiri and Laemmlen 1981). However, recent studies showed that several *Cytospora* species were associated with canker disease of spruce trees (Pan et al. 2018; Pan et al. 2021). The family Cytosporaceae was introduced by Fries in 1825, but it was synonymized under Valsaceae in 1861. Recently, the family name of Cytosporaceae was legitimated again (Rossman et al. 2015). The genus *Cytospora* was introduced by Ehrenberg in 1818 with four described species (*C.betulina*, *C.epimyces*, *C.resinae*, and *C.ribis*). Currently, the genus name has priority over *Leucocytospora*, *Leucostoma*, *Valsa*, *Valsella*, and *Valseutypella* based on the dual-nomenclature criterion (Adams et al. 2005; Rossman et al. 2015). The identification of *Cytospora* species was initially based on morphological characteristics and host affiliation. However, these criteria for species delimitation are not robust due to significant overlap in morphological traits and lack of host specificity. A systematic approach that combines ecological, morphological, and phylogenetic analyses is essential to identify, correctly describe, and name *Cytospora* species. Recent studies have supplied updated phylogenies for the genus using partial protein coding gene sequence data (*act1*, *rpb2*, *tef1-α*, *tub2*) with reference strains included in analyses (Hanifeh et al. 2022; Jia et al. 2024; Lin et al. 2024).

Since *Cytospora* species are found to be the main causal agents of spruce canker disease, more information is required to understand pathogen-host interactions and disease pathogenesis. In order to successfully colonize plant tissue, a fungus needs to overcome chemical and physical barriers established by a host. Fungal plant pathogens evolved different lifestyles (e.g., biotrophy), which require specific gene sets (de Wit et al. 2012). The gene contents of pathogenic fungi formed through gene family expansion or contraction events can be related to genome size and architecture (Raffaele and Kamoun 2011). The main gene families associated with pathogenesis include cell wall degrading enzymes (CWDE), biosynthesis gene clusters (BSGC), and effector proteins (Keller et al. 2005; Chang et al. 2016; Selin et al. 2016). The experimentally validated genes involved in the pathogen–host interactions can also be important pathogenicity determinants and virulence factors (Winnenburg et al. 2005). Comparative genomic analysis can provide insights into the molecular basis of pathogenicity mechanisms of *Cytospora* species isolated from affected trees. Such an analysis is usually performed for studied pathogenic species within a genus or a family (Wang et al. 2018; Yu et al. 2022; Liang et al. 2024). Considering that Diaporthales is a well-defined and diverse order of Sordariomycetes that comprises a number of plant pathogens (Rossman et al. 2007; Senanayake et al. 2017), the two introduced *Cytospora* genomes were included in a set of the diaporthalean species with publicly available genomic data.

Of the *Cytospora* genomes available via NCBI (http://www.ncbi.nlm.nih.gov/) and Mycocosm (Grigoriev et al. 2014), the strain *C.piceae* CFCC52841 (ex-type living culture) was previously reported as a pathogen of *Piceacrassifolia* (Pan et al. 2018). The genome was sequenced with a third-generation sequencing platform and analyzed within a set of six *Cytospora* species (Zhou et al. 2021). The performance in terms of quality between short and long-read sequencing has been broadly discussed (Miyamoto et al. 2014; Meslier et al. 2022). A comparison of two assemblies of *C.piceae* obtained from PacBio and Illumina reads will contribute to this discussion. The evidence will eventually help researchers with the selection of sequencing options. Genomic resources for another species isolated from diseased trees, *C.piceicola*, are unavailable.

This study identifies the species of *Cytospora* associated with canker disease of *Piceaglauca* (Moench) Voss in the Niagara Region, Ontario, Canada. The genomes of *C.piceae* EI-19(A) and novel species *C.piceicola* EI-20 were sequenced and analyzed to identify the contents of CWDEs, BSGCs, effectors, and virulence-associated genes. Additionally, a comparative analysis was performed to reveal the lifestyle and pathogenicity potential of the studied species. There is a noticeable lack of updated studies for the region. This absence hinders a comprehensive understanding of CC and its associated pathogens. Therefore, the results of this study will provide a foundation for further research of pathogenicity and virulence-related genes of fungal plant pathogens and formulate effective canker disease prevention and control strategies.

## ﻿Materials and methods

### ﻿Sample collection and pathogen isolation

Branches of *Piceaglauca* were collected from the trees with typical symptoms of CC in the Niagara region, Ontario, Canada, in 2020. The isolates were first obtained using the single spore isolation technique (Phookamsak et al. 2015). To isolate species of *Cytospora* from plant tissue, small pieces (0.5–1 cm) of cankered wood were surface sterilized with 70% ethanol for 30 s, following sterilization with 0.5% sodium hypochlorite for 2 min, rinsed three times with sterile water, and plated on malt extract agar (MEA). Purification of *Cytospora*-like colonies was performed by transferring hyphal tips to new MEA plates. The representative isolate obtained from necrotic plant tissue clearly resembling a *Cytospora* colony was selected for further analysis. The isolate EI-19(A) was identified based on morphological characteristics and sequence data analysis. To correctly identify the isolate EI-20, both detailed morphological and phylogenetic analyses were conducted. The specimens (branches with fruiting structures, wood samples, and living cultures) were deposited into the Herbarium of the Nature Research Centre (BILAS), Institute of Botany, Vilnius, Lithuania, the Canadian National Mycological Herbarium (DAOM), and the Canadian Collection of Fungal Cultures (DAOMC), Ottawa, Canada.

### ﻿Morphological analysis

The description of the new species was carried out using the pure culture of the representative isolate. Radial colony growth and color were assessed after 7 and 14 days of fungus incubation under room temperature in the dark on MEA, respectively. Morphological characterization and measurements of asexual reproductive structures (conidiomata (*n* = 10), conidiophores (*n* = 20), and conidia (*n* = 50)) were performed with dissecting (AmScope SE306R-PZ) and compound (AmScope B120C-E5) microscopes. Pictures were taken with a 12 MP digital AmScope camera MD1200A supplied with AmScopeX software (AmScope, Irvine, CA, USA).

### ﻿DNA extraction, PCR amplification, and sequencing

Total genomic DNA (gDNA) was extracted from 8-10-day-old pure cultures using the DNeasy PowerSoil Pro Kit (Qiagen, Hilden, Germany) following the manufacturer’s instructions. The internal transcribed spacer (ITS) region was amplified with the primer pair ITS1/ITS4 (White et al. 1990) using a C-1000 thermal cycler (Bio-Rad Laboratories, Hercules, CA, USA) under the conditions described in the references for the region. The quality of PCR products was examined using electrophoresis in 1% agarose gel. Sanger sequencing was carried out at the Genome Quebec Innovation Centre (Montreal, QC, Canada). The partial protein-coding gene (*act*, *rpb2*, *tef1-α*, *tub2*) sequence data were obtained from assemblies of the strains EI-19(A) and EI-20. The available sequences of the strain *C.piceae* CFCC 52842 were run against the strains’ genome assemblies to locate corresponding gene regions with the BLASTn search algorithm. The nucleotide sequences were further retrieved using BioEdit v.7.0 (Hall 1999).

### ﻿Sequence alignment and phylogenetic analysis

The initial identification was performed employing the BLASTn tool against the GenBank nucleotide database of the National Center for Biotechnology Information (NCBI). Sequence data of the related reference strains (Lin et al. 2024) were downloaded from the GenBank database. The sequences were initially aligned employing CLUSTAL-X2 v.2.1 (Thompson et al. 1994). Some characters were trimmed from both ends of the alignments to approximate the size of the obtained sequences to those included in the dataset with MEGA-X (Kumar et al. 2018). Phylogenetic analyses were executed using randomized accelerated maximum likelihood (RAxML) v. 8.0 (Stamatakis 2014) for maximum likelihood (ML) analysis. Bayesian posterior (BP) probabilities were defined using MrBayes v.3.2.7 (Huelsenbeck and Ronquist 2001) with the TrEase web server (Mishra et al. 2023). The ML analysis was performed using the general time-reversible (GTR) substitution model with a gamma-distributed rate of heterogeneity and a proportion of invariant sites selected with ModelTest-NG v.0.1.7 (Darriba et al. 2020). The statistical support values were estimated with bootstrapping of 1,000 replicates (Hillis and Bull 1993). The GTR model was also chosen for the BI analysis. The Markov chain Monte Carlo (MCMC) algorithm was used to estimate Bayesian posterior probabilities (BPP). Six simultaneous Markov chains were run for 1,000,000 generations. A burn-in was implemented by discarding the first 30% of generated trees. The phylograms were visualized using FigTree v. 1.4.4 (Rambaut 2018). The newly generated sequences were deposited in GenBank (Table 1). The master alignment used in the analyses was submitted to TreeBase (www.treebase.org; ID: S31874).

**Table 1.** Strains of the *Cytospora* genus used in phylogenetic analysis with their GenBank accession numbers. Ex-type strains are marked with ^T^. Strains obtained in this study are marked in bold. Sequence data retrieved from genome assemblies is marked with *. NA indicates that data is not available.

Species	Strain	Host	GenBank accession numbers				
			ITS	*act*	*rpb2*	*tef1-α*	*tub2*
*Cytosporaailanthicola*	CFCC 89970	*Ailanthusaltissima*	MH933618	MH933526	MH933592	MH933494	MH933565
*C.albodisca*	CFCC 53161	*Platycladusorientalis*	MW418406	MW422899	MW422909	MW422921	MW422933
*C.albodisca*	CFCC 54373	*Platycladisorientalis*	MW418407	MW422900	MW422910	MW422922	MW422934
*C.alba*	CFCC 55462^T^	*Salixmatsudana*	MZ702593	OK303457	OK303516	OK303577	OK303644
*C.alba*	CFCC 55463	*Salixmatsudana*	MZ702594	OK303458	OK303517	OK303578	OK303645
*C.ampulliformis*	MFLUCC 16-0583^T^	*Sorbusintermedia*	KY417726	KY417692	KY417794	NA	NA
*C.ampulliformis*	MFLUCC 16-0629	*Acerplatanoides*	KY417727	KY417693	KY417795	NA	NA
*C.amydgali*	CBS 144233^T^	*Prunusdulcis*	MG971853	MG972002	NA	MG971659	MG971718
*C.atrocirrhata*	CFCC 89615	*Juglansregia*	KR045618	KF498673	KU710946	KP310858	KR045659
*C.atrocirrhata*	CFCC 89616	*Juglansregia*	KR045619	KF498674	KU710947	KP310859	KR045660
*C.beilinensis*	CFCC 50493^T^	*Pinusarmandii*	MH933619	MH933527	NA	MH933495	MH933561
*C.beilinensis*	CFCC 50494	*Pinusarmandii*	MH933620	MH933528	NA	MH933496	MH933562
*C.berberidis*	CFCC 89927^T^	*Berberisdasystachya*	KR045620	KU710990	KU710948	KU710913	KR045661
*C.berberidis*	CFCC 89933	*Berberisdasystachya*	KR045621	KU710991	KU710949	KU710914	KR045662
*C.bungeana*	CFCC 50495^T^	*Pinusbungeanae*	MH933621	MH933529	MH933593	MH933497	MH933563
*C.bungeana*	CFCC 50496	*Pinusbungeanae*	MH933622	MH933530	MH933594	MH933498	MH933564
*C.californica*	CBS 144234^T^	*Juglansregia*	MG971935	MG972083	NA	MG971645	NA
*C.carbonacea*	CFCC 89947	*Ulmuspumila*	KR045622	KP310842	KU710950	KP310855	KP310825
*C.carpobroti*	CMW 48981^T^	*Carpobrotusedulis*	MH382812	NA	NA	MH411212	MH411207
*C.celtidicola*	CFCC 50497^T^	*Celtissinensis*	MH933623	MH933531	MH933595	MH933499	MH933566
*C.celtidicola*	CFCC 50498	*Celtissinensis*	MH933624	MH933532	MH933596	MH933500	MH933567
*C.centrivillosa*	MFLUCC 16-1206^T^	*Sorbusdomestica*	MF190122	NA	MF377600	NA	NA
*C.centrivillosa*	MFLUCC 17-1660	*Sorbusdomestica*	MF190123	NA	MF377601	NA	NA
*C.ceratosperma*	CFCC 89624	*Juglansregia*	KR045645	NA	KU710976	KP310860	KR045686
*C.ceratosperma*	CFCC 89625	*Juglansregia*	KR045646	NA	KU710977	KP31086	KR045687
*C.chrysosperma*	CFCC 89981	*Populusalba*	MH933625	MH933533	MH933597	MH933501	MH933568
*C.chrysosperma*	CFCC 89982	*Ulmuspumila*	KP281261	KP310835	NA	KP310848	KP310818
*C.cinnamomea*	CFCC 53178^T^	*Prunusarmeniaca*	MK673054	MK673024	NA	NA	MK672970
*C.coryli*	CFCC 53162^T^	*Corylusmandshurica*	MN854450	NA	MN850751	MN850758	MN861120
*C.corylina*	CFCC 54684^T^	*Corylusheterophylla*	MW839861	MW815951	MW815937	MW815886	MW883969
*C.corylina*	CFCC 54685	*Corylusheterophylla*	MW839862	MW815952	MW815938	MW815887	MW883970
*C.cotini*	MFLUCC 14-1050^T^	*Cotinuscoggygria*	KX430142	NA	KX430144	NA	NA
*C.cotoneastricola*	CF 20197027	*Cotoneaster* sp.	MK673072	MK673042	MK673012	MK672958	MK672988
*C.cotoneastricola*	CF 20197028	*Cotoneaster* sp.	MK673073	MK673043	MK673013	MK672959	MK672989
*C.curvispora*	CFCC 54000^T^	*Corylusheterophylla*	MW839851	MW815931	MW815945	MW815880	MW883963
*C.curvispora*	CFCC 54001	*Corylusheterophylla*	MW839854	MW815933	MW815947	MW815882	MW883965
*C.davidiana*	CXY 1350^T^	*Populusdavidiana*	KM034870	NA	NA	NA	NA
*C.diopuiensis*	CFCC55479	Undefined wood	MK912137	MN685819	NA	NA	NA
*C.diopuiensis*	CFCC55527	*Koelreuteriapaniculata*	ON376918	ON390905	ON390908	ON390914	ON390923
*C.discotoma*	CFCC 53137^T^	*Platycladusorientalis*	MW418404	MW422897	MW422907	MW422919	MW422931
*C.discotoma*	CFCC 54368	*Platycladusorientalis*	MW418405	MW422898	MW422908	MW422920	MW422932
*C.donetzica*	MFLUCC 15-0864	*Crataegusmonogyna*	KY417729	KY417695	KY417797	NA	NA
*C.donetzica*	MFLUCC 16-0574^T^	*Crataegusmonogyna*	KY417731	KY417697	KY417799	NA	NA
*C.donglingensis*	CFCC 53159^T^	*Platycladusorientalis*	MW418412	MW422903	MW422915	MW422927	MW422939
*C.donglingensis*	CFCC 53160	*Platycladusorientalis*	MW418414	MW422905	MW422917	MW422929	MW422941
*C.elaeagni*	CFCC 89632	*Elaeagnusangustifolia*	KR045626	KU710995	KU710955	KU710918	KR045667
*C.elaeagni*	CFCC 89633	*Elaeagnusangustifolia*	KF765677	KU710996	KU710956	KU710919	KR045668
*C.elaeagnicola*	CFCC 52882^T^	*Elaeagnusangustifolia*	MK732342	MK732345	MK732348	NA	NA
*C.elaeagnicola*	CFCC 52883	*Elaeagnusangustifolia*	MK732343	MK732346	MK732349	NA	NA
*C.erumpens*	CFCC 50022	*Prunuspadus*	MH933627	MH933534	NA	MH933502	MH933569
*C.erumpens*	CFCC 53163	*Prunuspadus*	MK673059	MK673029	MK673000	MK672948	MK672975
*C.eucalypti*	CBS 144241	*Eucalyptusglobulus*	MG971907	MG972056	NA	MG971617	MG971772
*C.euonymicola*	CFCC 50499^T^	*Euonymuskiautschovicus*	MH933628	MH933535	MH933598	MH933503	MH933570
*C.euonymicola*	CFCC 50500	*Euonymuskiautschovicus*	MH933629	MH933536	MH933599	MH933504	MH933571
*C.euonymina*	CFCC 89993^T^	*Euonymuskiautschovicus*	MH933630	MH933537	MH933600	MH933505	MH933590
*C.euonymina*	CFCC 89999	*Euonymuskiautschovicus*	MH933631	MH933538	MH933601	MH933506	MH933591
*C.fugax*	CXY 1371	*Populussimonii*	KM034852	NA	NA	NA	KM034891
*C.fugax*	CXY 1381	*Populusussuriensis*	KM034853	NA	NA	NA	KM034890
*C.galegicola*	MFLUCC 18-1199^T^	*Galegaofficinalis*	MK912128	MN685810	MN685820	NA	NA
*C.gigalocus*	CFCC 89620^T^	*Juglansregia*	KR045628	KU710997	KU710957	KU710920	KR045669
*C.gigalocus*	CFCC 89621	*Juglansregia*	KR045629	KU710998	KU710958	KU710921	KR045670
*C.gigaspora*	CFCC 50014	*Juniperusprocumbens*	KR045630	KU710999	KU710959	KU710922	KR045671
*C.gigaspora*	CFCC 89634^T^	*Salixpsammophila*	KF765671	KU711000	KU710960	KU710923	KR045672
*C.globosa*	MFLU 16-2054^T^	*Abiesalba*	MT177935	NA	MT432212	MT454016	NA
*C.globosa*	CBS 118977	*Abiesalba*	PP988852	KX964768	KX965518	KX965130	KX964947
*C.granati*	CBS 144237^T^	*Punicagranatum*	MG971799	MG971949	NA	MG971514	MG971664
*C.haidianensis*	CFCC 54056	*Euonymusalatus*	MT360041	MT363978	MT363987	MT363997	MT364007
*C.haidianensis*	CFCC 54057^T^	*Euonymusalatus*	MT360042	MT363979	MT363988	MT363998	MT364008
*C.hejingensis*	CFCC 59571^T^	*Salix* sp.	PP060455	PP059657	PP059663	PP059667	PP059673
*C.hejingensis*	C3479	*Salix* sp.	PP060456	PP059658	PP059664	PP059668	PP059674
*C.hippophaës*	CFCC 89639	*Hippophaërhamnoides*	KR045632	KU711001	KU710961	KU710924	KR045673
*C.hippophaës*	CFCC 89640	*Hippophaërhamnoides*	KF765682	KF765730	KU710962	KP310865	KR045674
*C.japonica*	CFCC 89956	*Prunuscerasifera*	KR045624	KU710993	KU710953	KU710916	KR045665
*C.japonica*	CFCC 89960	*Prunuscerasifera*	KR045625	KU710994	KU710954	KU710917	KR045666
*C.jilongensis*	CFCC 59569^T^	*Prunusdavidiana*	PP060457	PP059659	NA	PP059669	PP059675
*C.jilongensis*	XZ083	*Prunusdavidiana*	P060458	PP059660	NA	PP059670	PP059676
*C.joaquinensis*	CBS 144235	*Populusdeltoides*	MG971895	MG972044	NA	MG971605	MG971761
*C.junipericola*	MFLU 17-0882^T^	*Juniperuscommunis*	MF190125	NA	NA	MF377580	NA
*C.juniperina*	CFCC 50501^T^	*Juniperusprzewalskii*	MH933632	MH933539	MH933602	MH933507	NA
*C.juniperina*	CFCC 50502	*Juniperusprzewalskii*	MH933633	MH933540	MH933603	MH933508	MH933572
*C.kantschavelii*	CXY 1383	*Populusmaximowiczii*	KM034867	NA	NA	NA	NA
*C.kuanchengensis*	CFCC 52464^T^	*Castaneamollissima*	MK432616	MK442940	MK578076	NA	NA
*C.kuanchengensis*	CFCC 52465	*Castaneamollissima*	MK432617	MK442941	MK578077	NA	NA
*C.kunsensis*	CFCC 59570^T^	*Prunuspadus*	PP060459	PP059661	PP059665	PP059671	PP059677
*C.kunsensis*	C3488	*Prunuspadus*	PP060460	PP059662	PP059666	PP059672	PP059678
*C.leucosperma*	CFCC 89622	*Pyrusbretschneideri*	KR045616	KU710988	KU710944	KU710911	KR045657
*C.leucosperma*	CFCC 89894	*Pyrusbretschneideri*	KR045617	KU710989	KU710945	KU710912	KR045658
*C.longispora*	CBS 144236^T^	*Prunusdomestica*	MG971905	MG972054	NA	MG971615	MG971764
*C.longistiolata*	MFLUCC 16-0628	Salix×fragilis	KY417734	KY417700	KY417802	NA	NA
*C.lumnitzericola*	MFLUCC 17-0508^T^	*Lumnitzeraracernosa*	MG975778	MH253457	MH253453	NA	NA
*C.mali*	CFCC 50028	*Maluspumila*	MH933641	MH933548	MH933606	MH933513	MH933577
*C.mali*	CFCC 50029	*Maluspumila*	MH933642	MH933549	MH933607	MH933514	MH933578
*C.mali-spectabilis*	CFCC 53181^T^	*Malusspectabilis*	MK673066	MK673036	MK673006	MK672953	MK672982
*C.melnikii*	CFCC 89984	*Rhustyphina*	MH933678	MH933551	MH933609	MH933515	MH933580
*C.mougeotii*	ATCC 44994^T^	*Piceaabies*	AY347329	NA	NA	NA	NA
*C.mougeotii*	CBS 198.50	*Piceaabies*	PP988918	KX964794			
*C.myrtagena*	CFCC 52454	*Castaneamollissima*	MK432614	MK442938	MK578074	NA	NA
*C.myrtagena*	CFCC 52455	*Castaneamollissima*	MK432615	MK442939	MK578075	NA	NA
*C.nivea*	CFCC 89641	*Elaeagnusangustifolia*	KF765683	KU711006	KU710967	KU710929	KR045679
*C.nivea*	MFLUCC 15-0860	*Salixacutifolia*	KY417737	KY417703	KY417805	NA	NA
*C.notastroma*	NE_TFR5	*Populustremuloides*	JX438632	NA	NA	JX438543	NA
*C.notastroma*	NE_TFR8	*Populustremuloides*	JX438633	NA	NA	JX438542	NA
*C.ochracea*	CFCC 53164^T^	*Cotoneaster* sp.	MK673060	MK673030	MK673001	MK672949	MK672976
*C.oleicola*	CBS 144248^T^	*Oleaeuropaea*	MG971944	MG972098	NA	MG971660	MG971752
*C.olivacea*	CFCC 53174	*Prunuscerasifera*	MK673058	MK673028	MK672999	NA	MK672974
*C.olivacea*	CFCC 53175	*Prunusdulcis*	MK673062	MK673032	MK673003	NA	MK672978
*C.palm*	CXY 1276	*Cotinuscoggygria*	JN402990	NA	NA	KJ781296	NA
*C.palm*	CXY 1280^T^	*Cotinuscoggygria*	JN411939	NA	NA	KJ781297	NA
*C.parakantschavelii*	MFLUCC 15-0857^T^	Populus×sibirica	KY417738	KY417704	KY417806	v	
*C.paracinnamomea*	CFCC 55453	*Salixmatsudana*	MZ702594	OK303456	OK303515	OK303576	OK303643
*C.paracinnamomea*	CFCC 55455^T^	*Salixmatsudana*	MZ702598	OK303460	OK303519	OK303580	OK303647
*C.parapistaciae*	CBS 144506^T^	*Pistaciavera*	MG971804	MG971954	NA	MG971519	MG971669
*C.paraplurivora*	FDS-439	*Prunusarmeniaca*	OL640182	OL631586	NA	OL631589	NA
*C.paraplurivora*	FDS-564^T^	*Prunuspersica*	OL640183	OL631587	NA	OL631590	NA
*C.parasitica*	CFCC 53173	*Berberis* sp.	MK673070	MK673040	MK673010	MK672957	MK672986
*C.paratranslucens*	MFLUCC 15-0506^T^	Populusalbavar.bolleana	KY417741	KY417707	KY417809	NA	NA
*C.paratranslucens*	MFLUCC 16-0627	*Populusalba*	KY417742	KY417708	KY417810	NA	NA
*C.phialidica*	MFLUCC 17-2498	*Alnusglutinosa*	MT177932	NA	MT432209	MT454014	NA
*C.piceae*	CFCC 52841^T^	*Piceacrassifolia*	MH820398	MH820406	MH820395	MH820402	MH820387
*C.piceae*	CFCC 52842	*Piceacrassifolia*	MH820399	MH820407	MH820396	MH820403	MH820388
***C.piceae***	**EI-19(A), BILAS 51883**	***Piceaglauca***	**ON352564**	genome*	genome*	genome*	genome*
***C.piceicola***	**EI-20, BILAS 51886^T^**	***Piceaglauca***	**ON352567**	genome*	genome*	genome*	genome*
*C.pinastri*	CBS 113.81	*Abiesalba*	KY051777	KX964689	NA	NA	KX964886
*C.pinastri*	CBS 505.7	*Abiesalba*	KY051939	KX964819	NA	NA	KX964992
*C.pingbianensis*	MFLUCC 18-1204^T^	Undefined wood	MK912135	MN685817	MN685826	NA	NA
*C.pistaciae*	CBS 144238^T^	*Pistaciavera*	MG971802	MG971952	NA	MG971517	MG971667
*C.platyclade*	CFCC 50504^T^	*Platycladusorientalis*	MH933645	MH933552	MH933610	MH933516	MH933581
*C.platyclade*	CFCC 50505	*Platycladusorientalis*	MH933646	MH933553	MH933611	MH933517	MH933582
*C.platycladicola*	CFCC 50038^T^	*Platycladusorientalis*	KT222840	MH933555	MH933613	MH933519	MH933584
*C.platycladicola*	CFCC 50039	*Platycladusorientalis*	KR045642	KU711008	KU710973	KU710931	KR045683
*C.plurivora*	CBS 144239^T^	*Oleaeuropaea*	MG971861	MG972010	NA	MG971572	MG971726
*C.populi*	CFCC 55472^T^	*Populus* sp.	MZ702609	OK303471	OK303530	OK303591	OK303658
*C.populi*	CFCC 55473	*Populus* sp.	MZ702610	OK303472	OK303531	OK303592	OK303659
*C.populicola*	CBS 144240	*Populusdeltoides*	MG971891	MG972040	NA	MG971601	MG971757
*C.populina*	CFCC 89644^T^	*Salixpsammophila*	KF765686	KU711007	KU710969	KU710930	KR045681
*C.populinopsis*	CFCC 50032^T^	*Sorbusaucuparia*	MH933648	MH933556	MH933614	MH933520	MH933585
*C.populinopsis*	CFCC 50033	*Sorbusaucuparia*	MH933649	MH933557	MH933615	MH933521	MH933586
*C.predappioensis*	MFLUCC 17-2458^T^	*Platanushybrida*	MG873484	NA	NA	NA	NA
*C.pruinopsis*	CFCC 50034^T^	*Ulmuspumila*	KP281259	KP310836	KU710970	KP310849	KP310819
*C.pruinopsis*	CFCC 53153	*Ulmuspumila*	MN854451	MN850763	MN850752	MN850759	MN861121
*C.pruinosa*	CFCC 50036	*Syringaoblata*	KP310800	KP310832	NA	KP310845	KP310815
*C.pruinosa*	CFCC 50037	*Syringaoblata*	MH933650	MH933558	NA	MH933522	MH933589
*C.prunicola*	MFLU 17-0995^T^	*Prunus* sp.	MG742350	MG742353	MG742352	NA	NA
*C.pruni-mume*	CFCC 53179	*Prunusarmeniaca*	MK673057	MK673027	NA	MK672947	MK672973
*C.pruni-mume*	CFCC 53180^T^	*Prunusmume*	MK673067	MK673037	MK673007	MK672954	MK672983
*C.quercicola*	MFLU 17-0881	*Quercus* sp.	MF190128	NA	NA	NA	NA
*C.ribis*	CFCC 50026	*Ulmuspumila*	KP281267	KP310843	KU710972	KP310856	KP310826
*C.ribis*	CFCC 50027	*Ulmuspumila*	KP281268	KP310844	NA	KP310857	KP310827
*C.rosicola*	CF 20197024^T^	*Rosa* sp.	MK673079	MK673049	MK673019	MK672965	MK672995
*C.rostrata*	CFCC 89909	*Salixcupularis*	KR045643	KU711009	KU710974	KU710932	KR045684
*C.rostrata*	CFCC 89910	*Salixcupularis*	KR045644	KU711010	KU710975	KU710933	NA
*C.rusanovii*	MFLUCC 15-0853	Populus×sibirica	KY417743	KY417709	KY417811	NA	NA
*C.rusanovii*	MFLUCC 15-0854^T^	*Salixbabylonica*	KY417744	KY417710	KY417812	NA	NA
*C.saccardoi*	CBS 109752	*Juniperuscommunis*	PP988975	KX964683	KX965461	KX965050	KX964883
*C.saccardoi*	CBS 141615	Unknown	PP988976	NA	NA	NA	NA
*C.sacculus*	CFCC 89626^T^	*Juglansregia*	KR045647	KU711011	KU710978	KU710934	KR045688
*C.sacculus*	CFCC 89627	*Juglansregia*	KR045648	KU711012	KU710979	KU710935	KR045689
*C.salicacearum*	MFLUCC 15-0509	*Salixalba*	KY417746	KY417712	KY417814	NA	NA
*C.salicacearum*	MFLUCC 15-0861	Salix×fragilis	KY417745	KY417711	KY417813	NA	NA
*C.salicicola*	MFLUCC 14-1052^T^	*Salixalba*	KU982636	KU982637	NA	NA	NA
*C.salicicola*	MFLUCC 15-0866	*Salix* sp.	KY417749	KY417715	KY417817	NA	NA
*C.salicina*	MFLUCC 15-0862	*Salixalba*	KY417750	KY417716	KY417818	NA	NA
*C.salicina*	MFLUCC 16-0637	Salix×fragilis	KY417751	KY417717	KY417819	NA	NA
*C.schulzeri*	CFCC 50042	*Maluspumila*	KR045650	KU711014	KU710981	KU710937	KR045691
*C.sibiraeae*	CFCC 50045^T^	*Sibiraeaangustata*	KR045651	KU711015	KU710982	KU710938	KR045692
*C.sibiraeae*	CFCC 50046	*Sibiraeaangustata*	KR045652	KU711015	KU710983	KU710939	KR045693
*C.sophorae*	CFCC 50047	*Styphnolobiumjaponicum*	KR045653	KU711017	KU710984	KU710940	KR045694
*C.sophorae*	CFCC 89598	*Styphnolobiumjaponicum*	KR045654	KU711018	KU710985	KU710941	KR045695
*C.sophoricola*	CFCC 89595^T^	*Styphnolobiumjaponicum*	KR045655	KU711019	KU710986	KU710942	KR045696
*C.sophoricola*	CFCC 89596	*Styphnolobiumjaponicum*	KR045656	KU711020	KU710987	KU710943	KR045697
*C.sophoriopsis*	CFCC 55489	*Salixmatsudana*	MZ702583	OK303445	OK303504	OK303565	OK303632
*C.sophoriopsis*	CFCC 89600	*Styphnolobiumjaponicum*	KR045623	KU710992	KU710951	KU710915	KP310817
*C.sorbi*	MFLUCC 16-0631^T^	*Sorbusaucuparia*	KY417752	KY417718	KY417820	NA	NA
*C.sorbicola*	MFLUCC 16-0584^T^	*Acerpseudoplatanus*	KY417755	KY417721	KY417823	NA	NA
*C.sorbicola*	MFLUCC 16-0633	*Cotoneastermelanocarpus*	KY417758	KY417724	KY417826	NA	NA
*C.sorbina*	CF 20197660^T^	*Sorbustianschanica*	MK673052	MK673022	NA	MK672943	MK672968
*C.spiraeae*	CFCC 50049^T^	*Spiraeasalicifolia*	MG707859	MG708196	MG708199	NA	NA
*C.spiraeae*	CFCC 50050	*Spiraeasalicifolia*	MG707860	MG708197	MG708200	NA	NA
*C.spiraeicola*	CFCC 53138^T^	*Spiraeasalicifolia*	MN854448	NA	MN850749	MN850756	MN861118
*C.spiraeicola*	CFCC 53139	*Tilianobilis*	MN854449	NA	MN850750	MN850757	MN861119
*C.tamaricicola*	CFCC 50507	*Rosamultifolora*	MH933651	MH933559	MH933616	MH933525	MH933587
*C.tamaricicola*	CFCC 50508^T^	*Tamarixchinensis*	MH933652	MH933560	MH933617	MH933523	MH933588
*C.tanaitica*	MFLUCC 14-1057^T^	*Betulapubescens*	KT459411	KT459413	NA	NA	NA
*C.thailandica*	MFLUCC 17-0262^T^	*Xylocarpusmoluccensis*	MG975776	MH253459	MH253455	NA	NA
*C.thailandica*	MFLUCC 17-0263	*Xylocarpusmoluccensis*	MG975777	MH253460	MH253456	NA	NA
*C.tibetensis*	CF 20197026	*Cotoneaster* sp.	MK673076	MK673046	MK673016	MK672962	MK672992
*C.tibetensis*	CF 20197029	*Cotoneaster* sp.	MK673077	MK673047	MK673017	MK672963	MK672993
*C.tibouchinae*	CPC 26333^T^	*Tibouchinasemidecandra*	KX228284	NA	NA	NA	NA
*C.translucens*	CXY 1351	*Populusdavidiana*	KM034874	NA	NA	NA	KM034895
*C.translucens*	CXY 1359	Populus×beijingensis	KM034871	NA	NA	NA	KM034894
*C.ulmi*	MFLUCC 15-0863^T^	*Ulmusminor*	KY417759	NA	NA	NA	NA
*C.verrucosa*	CFCC 53157^T^	*Platycladusorientalis*	MW418408	NA	MW422911	MW422923	MW422935
*C.verrucosa*	CFCC 53158	*Platycladusorientalis*	MW418410	MW422901	MW422913	MW422925	MW422937
*C.vinacea*	CBS 141585^T^	*Vitisinterspecific*	KX256256	NA	NA	KX256277	KX256235
*C.viridistroma*	CBS 202.36^T^	*Cerciscanadensis*	MN172408	NA	NA	MN271853	NA
*C.viticola*	Cyt2	*Vitisinterspecific*	KX256238	NA	NA	KX256259	KX256217
*C.viticola*	CBS 141586^T^	*Vitisvinifera*	KX256239	NA	NA	KX256260	KX256218
*C.xinjiangensis*	CFCC 53182	*Rosa* sp.	MK673064	MK673034	MK673004	MK672951	MK672980
*C.xinjiangensis*	CFCC 53183^T^	*Rosa* sp.	MK673065	MK673035	MK673005	MK672952	MK672981
*C.xinglongensis*	CFCC 52458^T^	*Castaneamollissima*	MK432622	MK442946	MK578082	NA	NA
*C.xinglongensis*	CFCC 52459	*Castaneamollissima*	MK432623	MK442947	MK578083	NA	NA
*C.xylocarpi*	MFLUCC 17-0251^T^	*Xylocarpusgranatum*	MG975775	MH253458	MH253454	NA	NA
*C.zhaitangensis*	CFCC 56227^T^	*Euonymusjaponicus*	OQ344750	OQ398760	OQ398789	OQ410623	OQ398733
*C.zhaitangensis*	CFCC 57537	*Euonymusjaponicus*	OQ344751	OQ398761	OQ398790	OQ410624	OQ398734
*Diaporthevaccinii*	CBS 160.32	*Vacciniummacrocarpon*	KC343228	JQ807297	NA	KC343954	KC344196

### ﻿Library preparation, genome sequencing, and assembly

The obtained gDNA of the strains EI-19(A) and EI-20 was quantified employing the Quant-iT PicoGreen dsDNA Assay Kit (Life Technologies, Carlsbad, CA, USA). Libraries were prepared with the NEBNext Ultra II DNA Library Preparation Kit for Illumina (New England Biolabs, Ipswich, MA, USA). Whole genome sequencing was performed using the NovaSeq 6000 platform with a 150 bp pair-end sequencing strategy. This option was selected as being more cost-effective for obtaining draft genome sequences compared to third-generation sequencing. All procedures were performed at the Genome Quebec Innovation Centre (Montreal, Canada). The raw reads were quality-filtered using Trimmomatic v. 0.38.1 (Bolger et al. 2014) with the implementation of Nextera (pair-ended) and sliding window settings. The obtained reads were assembled into scaffolds using SPAdes v. 3.14.1 with K-mer values 21, 33, 45, 57, 69, 81, 93, 105, and 117. The genome assembly characteristics were assessed using QUAST v. 5.2.0 (Mikheenko et al. 2018). Repeat sequence content was estimated with RepeatMasker v. 4.1.5 (Smit et al. 2013), employing the Repbase library of repeats for fungi (Bao et al. 2015). The tRNA regions were predicted using the tRNA scan tool v. 0.4 (Lowe and Eddy 1997). The completeness of assembly was estimated with BUSCO v.5.7.1 (Simão et al. 2015) based on the dataset ascomycota_odb10 (Manni et al. 2021).

### ﻿Gene prediction and functional annotation

Gene prediction from assemblies of the strains EI-19(A) and EI-20 was performed using Augustus v. 3.4.0 (Stanke and Morgenstern 2005) with the gene models of *C.mali* 03-8 employed to train the tool. For consistency, the genes of other strains included in the analysis were predicted using *Fusariumgraminearum* as a model organism with default parameters. The eggNOG mapper tool v. 2.1.9 (Huerta-Cepas et al. 2019) was employed for functional annotation of the genes predicted. The annotation of CWDEs was performed using dbCAN3 (Yin et al. 2012) with the HMMER-based classification tool. BSGCs were predicted with antiSMASH v. 6.1.1 (Blin et al. 2021). Secretomes were identified using the SECRETOOL pipeline (Cortázar et al. 2014) with default cut-off values and further classified (Miyauchi et al. 2020). EffectorP-fungi v.3.0 (Sperschneider and Dodds 2022) was employed to predict effector proteins. The PHI-base dataset v. 4.17 (Urban et al. 2017), containing 9,056 protein sequences, was used to identify virulence-related genes. A local BLASTp search was performed based on the following parameters: *e*-value 1 × 10^−5^, minimum query coverage per HSP 100%, and percent identity cut off ≥ 65%.

### ﻿Phylogenetic tree reconstruction, genome alignment, divergence time estimation, and gene family analysis

The single-copy orthogroups (SCOs) within all species included in the analysis were identified with Orthofinder v. 2.5.4 (Emms and Kelly 2015). Multiple sequence alignment was performed using MAFFT v. 7.310 (Katoh and Standley 2013). ModelFinder (Kalyaanamoorthy et al. 2017) was used to select a model for each alignment. The phylogenetic tree was produced based on concatenated alignments employing IQ-Tree v. 2.1.3 (Nguyen et al. 2015). Genome sequences were aligned with MashMap v. 2.0 (Jain et al. 2018) and displayed as dot-plot graphs using D-Genies (Cabanettes and Klopp 2018). The RealTime tool implemented in MEGA-X (Kumar et al. 2018) was employed to estimate divergence times. The secondary calibration nodes for the splits of *Juglanconisjuglandina* and *Diaporthecitrichinensis* (mean: 55.9 MYA, standard deviation: 1 MYA) and *Diaporthecitrichinensis* and *Cytosporachrysosperma* (mean: 42.3 MYA, standard deviation: 1 MYA) were used for divergence time estimations based on the TimeTree dataset (Kumar et al. 2017). Gene family expansion and contraction events were revealed using CAFE5 (Mendes et al. 2020) with a *p*-value of 0.01 as the threshold.

## ﻿Results and discussion

### ﻿Phylogenetic analyses

**Figure 2.**
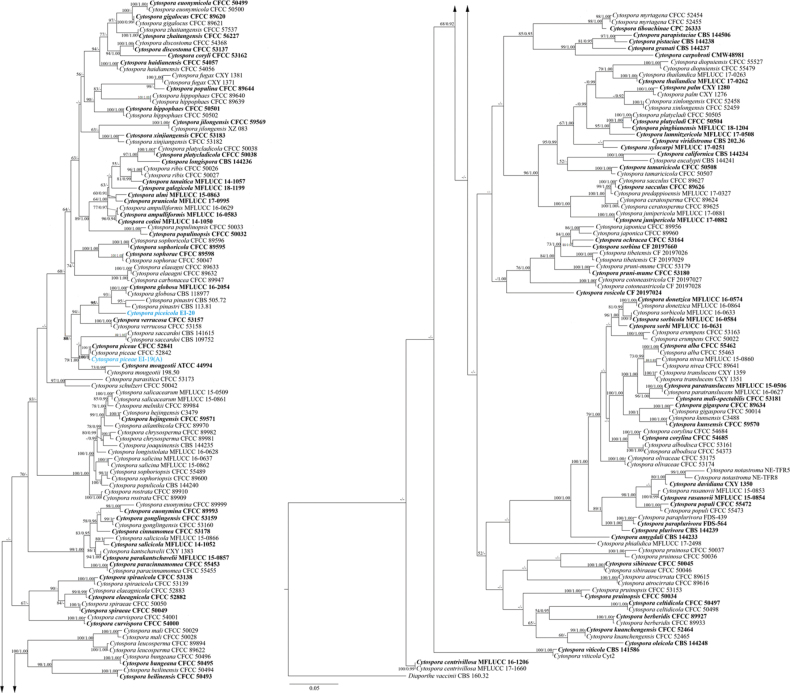
Phylogram of the RAxML tree generated based on the analysis of combined ITS, *act*, *rpb2*, *tef1-α*, and *tub2* sequence data of the *Cytospora* genus. Bootstrap support values for ML ≥ 50% and BP ≥ 0.90 are shown as ML/BP above or below the nodes. Ex-type strains are in bold. Strains obtained in this study are in blue. The tree is rooted to *Diaporthevaccinii* (CBS 160.32).

The ML and BP analyses of the combined ITS, *act*, *rpb2*, *tef1-α*, and *tub2* sequence data produced phylogenetic trees with highly similar topologies. The best-scoring ML tree with a log-likelihood value of −63932.107826 is shown in Fig. 2. Estimated base frequencies were as follows: A = 0.245570, C = 0.2245652, G = 0.261603, T = 0.247175; substitution rates: AC = 1.770329, AG = 2.617916, AT = 1.724382, CG = 1.002700, CT = 5.573700, GT = 1.000000. The strain of the novel species *C.piceicola* EI-20 was nested separately, forming a well-supported clade with *C.globosa* and *C.pinastri* (95% ML). Notably, *C.piceicola* was also grouped (80% ML) with other species isolated from conifer trees (*C.mougeotii*, *C.piceae*, *C.saccardoi*, and *C.verrucosa*).

### ﻿Taxonomy

**Cytosporaceae Fr., Systema Orbis Vegetabilis 1: 118 (1825)**

***Cytospora* Ehrenb., Sylvae mycologicae Berolinenses: 28 (1818)**

#### ﻿ Cytospora piceae

Taxon classificationFungiDiaporthalesCytosporaceae

X.L. Fan, in Pan, Zhu, Tian, Alvarez & Fan, Phytotaxa 383(2): 188 (2018)

87A01DB9-CFFB-52D3-9480-4B8930E38F4E

Fig. 3

##### Description.

***Sexual morph***: not observed. ***Asexual morph*: *Conidiomata*** pycnidial, immersed in bark, erumpent, ostiolated, with multiple irregularly arranged circular or ovoid locules, 1,050–1,400 μm in diam. Conceptacle absent, ostiole conspicuous, circular, dark gray, at the same level as the disc. ***Conidiophores*** semimacronematous, hyaline, filamentous, mainly unbranched or branched at base, elongated, smooth, thin-walled, (16.0–)17.7–22.2(–23.5) μm. ***Conidiogenous cells*** enteroblastic, polyphialidic. ***Conidia*** abundant, single, hyaline, aseptate, curved, allantoid, thin-walled (5.0–)5.4–6.1(–6.5) × (1.0–)1.2–1.5 μm.

**Figure 3.**
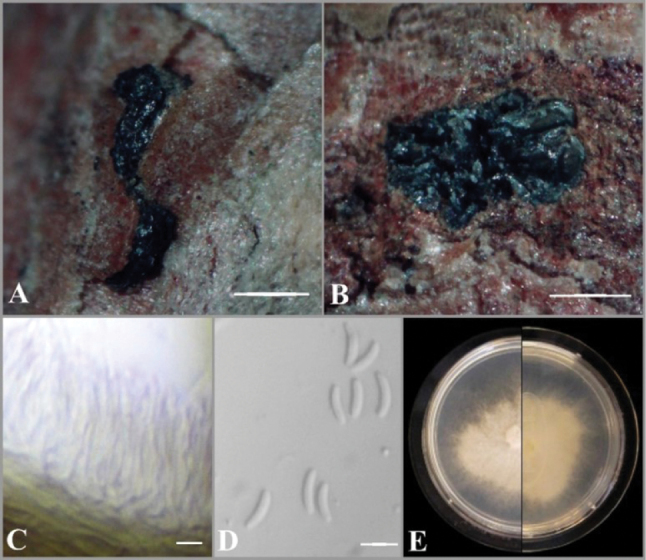
Asexual morph of *Cytosporapiceae***A** habit of conidiomata on twig of *P.glauca***B** transverse section of conidioma **C** conidiophores and conidiogenous cells **D** conidia **E** adverse and reverse view of seven-day-old culture on MEA. Scale bars: 1 mm (**A, B**); 5 μm (**C, D**).

##### Culture characteristics.

*Colonies* on MEA initially white, becoming beige with dense aerial mycelium, slow-growing (17 mm in diameter) after 7 days of incubation. *Hyphae* hyaline, smooth, branched, and septate.

##### Material examined.

Canada • Ontario, Lincoln, 43°06'38.2"N, 79°19'17.5"W, branches of *Piceaglauca* (Moench) Voss, pycnidia (conidiomata) formed on cankered branches and twigs, April 2020, E. Ilyukhin (BILAS 51883, DAOM 985023, DAOMC 256987).

##### Notes.

Morphologically, two isolates of *C.piceae* are very similar, but EI-19(A) has slightly longer conidiophores and conidia than CFCC 52841. Originally, *C.piceae* was described as a pathogen associated with the canker disease of *Piceacrassifolia* in China (Pan et al. 2018). But the species was isolated from different hosts (including non-conifers) in other parts of Canada based on ITS sequence data (ON352565, PQ671332, PQ671333, PQ666762) available in GenBank.

#### ﻿ Cytospora piceicola

Taxon classificationFungiDiaporthalesCytosporaceae

Ilyukhin & Markovsk. sp. nov.

0E5E70CD-2F46-56B6-8837-A111A55E8EFB

856480

Fig. 4

##### Etymology.

The name refers to the host genus, *Picea*, from which the fungus was first isolated.

**Figure 4.**
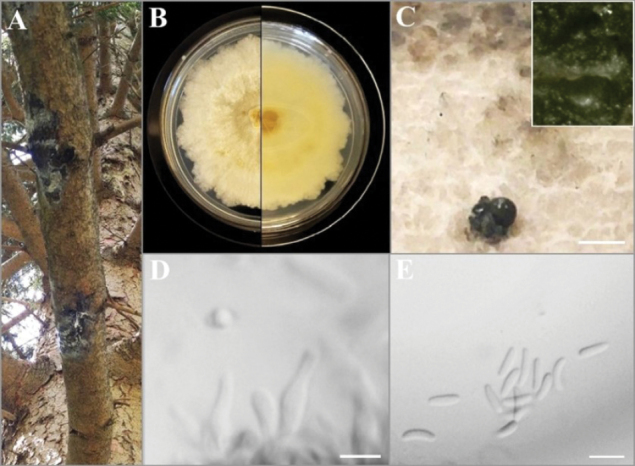
Asexual morph of *C.piceicola***A** isolation source (cankered branches of *P.glauca*) **B** adverse and reverse view of seven-day-old culture on MEA**C** pycnidia (with locules) on the surface of the colony after 25 days of incubation **D** conidiogenous cells **E** conidia. Scale bars: 1 mm (**C**); 5 μm (**D, E**).

##### Holotype.

Canada • Ontario, Lincoln, 43°06'39.0"N, 79°19'15.4"W, isolated from cankered wood (branches) of *Piceaglauca*, April 2020, E. Ilyukhin (holotype BILAS 51884, ex-holotype living culture BILAS 51886=EI-20, isotype DAOM 985024, DAOMC 256985).

##### Description.

***Sexual morph***: not observed in culture. ***Asexual morph*: *Conidiomata*** appearing after 25 days of incubation on MEA, rare, pycnidial, solitary, globose to subglobose, dark grey to black when dry, with few ovoid locules, (610–)824–1071(–1380) μm diam. ***Conidiophores*** micronematous, hyaline, smooth-walled, reduced to unbranched conidiogenous cells. ***Conidiogenous cells*** enteroblastic, phialidic, lageniform, or ampulliform (7.5–)8.8–10.6(–13.0) × (1.0–)1.3–1.7(–2.0) μm. ***Conidia*** abundant, relatively small, single, hyaline, aseptate, slightly curved, allantoid, thin-walled (3.5–)3.8–4.9(–5.5) × (1.0–)0.8–1.3(–1.5) μm.

##### Culture characteristics.

*Colonies* on MEA white to light brown with short aerial mycelium tufts in the center, becoming darker with age, relatively slow-growing (28 mm in diameter) after 7 days of incubation. *Hyphae* hyaline, smooth, branched, and septate.

##### Notes.

Based on ITS sequence data, *C.piceicola* is 99% similar to *C.globosa* MFLU:16-2054 (554/559, 3 gaps) and *C.pinastri* CBS 113.81 (540/545, 0 gaps). But combined multi-gene phylogenetic analysis clearly distinguished *C.piceicola* from these two species (ML/BI = 95/-). The new species, *C.piceicola*, differs from *C.globosa* (4–6.5 × 1–2 µm) and *C.pinastri* (4–7 × 1–1.3 μm) by having shorter conidia and clearly lageniform or ampulliform conidiogenous cells (Hayova and Minter 2013; Li et al. 2020). The culture characteristics cannot be used for species discrimination as they are either unavailable (*C.pinastri*) or different media has been used (*C.globosa*). Thus, *C.piceicola* is considered a novel species based on both molecular data and morphological characteristics.

### ﻿Synteny analysis and genome assembly characteristics

**Figure 5.**
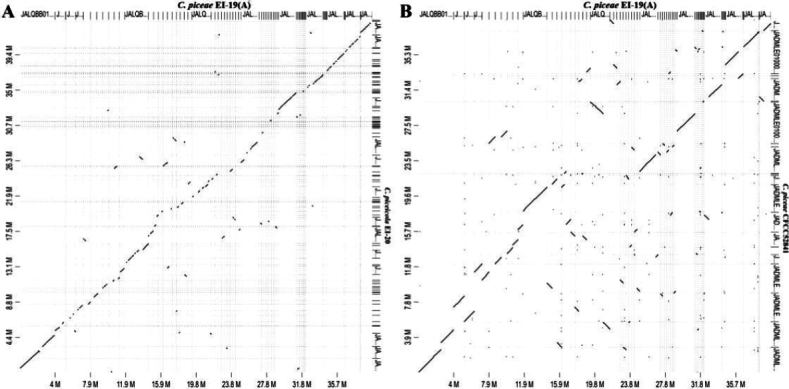
Dot-plot diagram showing genome sequence alignments of *C.piceae* EI-19(A) and *C.piceicola* EI-20(**A**), and *C.piceae* EI-19(A) and *C.piceae* CFCC 52841(**B**).

Synteny analysis employed in comparative genomics is crucial to understanding molecular-level similarities and differences in species diversity and genome evolution (He et al. 2023). Despite the close phylogenetic relationship between the studied species and the same sequencing strategy used, only 49.08% of synteny blocks in *C.piceae* EI-19(A) matched those in *C.piceicola* EI-20 with minor gaps and few inversions (Fig. 5A). The low-scale synteny linked to genome rearrangements might facilitate species-specific evolution of pathogenicity-related genes and contribute to ecological niche adaptation (e.g., host switch) (Sillo et al. 2015; Zeng et al. 2017). The analysis between assemblies of two strains of *C.piceae* revealed that there was no match for 6.51% of aligned sequences while 93.49% of them were more than 75% similar with large gaps, multiple inversions, and some repeats (Fig. 5B). It indicated a high degree of homology between the two genomes, typical for different strains of the same species (Hao et al. 2021). However, some assembly characteristics of the *C.piceae* strains appeared to differ (Table 2). The genome of *C.piceae* CFCC 52841 consisted of 21 scaffolds with N50 of 2.94 Mbp, which indicated better assembly quality than *C.piceae* EI-19(A) (105 scaffolds with N50 of 1.22). In addition, the assembly of the former contained notably fewer tRNAs (176) compared to *C.piceae* EI-19(A) (203). 8.82% of repeat classes were detected in the assembly of *C.piceae* CFCC 52841 based on multiple repeat masking tools (Zhou et al. 2021). This is higher than the average repeat content (5.18%) revealed for a large set of ascomycete genomes sequenced with an Illumina platform (Yu et al. 2022). A nearly complete (99.7%) genome assembly was reported for the strain *C.piceae* CFCC 52841 based on the fungi_odb9 dataset (Zhou et al. 2021). When employing the more specific ascomycota_odb10 dataset (Manni et al. 2021), assemblies of both strains were found to have lower completeness (*C.piceae* EI-19(A) (97.6%), *C.piceae* CFCC52841 (97.4%)). The genome of *C.piceicola* EI-20 was 98.1% complete. Among the analyzed Cytosporaceae species, the assembly of *C.chrysosperma* CFL2056 sequenced with a PacBio platform was 93.7% complete, whereas the completeness of the *C.leucostoma* SXYLt’s genome obtained with Illumina short reads was 98.6%.

**Table 2.** Genome assembly statistics of *C.piceae* EI-19(A), *C.piceae* CFCC 52841, and *C.piceicola* EI-20.

Assembly Features	*C.piceae* EI-19(A)	*C.piceae* CFCC 52841	*C.piceicola* EI-20
Genome size	39.7	39.2	43.8
Genome coverage (×)	217	200	195
Scaffolds (>1000 bp)	105	21	130
Scaffold N50 (Mb)	1.22	2.94	0.81
GC content (%)	51.37	51.79	49.11
N of genes predicted	10,862	10,835	10,742
Repeat rate (%)	2.29	2.62*	3.15
tRNAs	203	176	207
BUSCO estimates (%)	97.6	97.4*	98.1

* Based on analyses performed in this study.

Another study reported similar BUSCO estimates (97.8%–99.2%) for a set of the eleven *Diaporthe* species assemblies obtained with both the second and third-generation sequencing technologies (Hilário et al. 2022). Considering some limitations such as higher error rates (Alkan et al. 2011; Stoler and Nekrutenko 2021), Illumina technology can still produce high-quality reads, allowing for the assembly of nearly complete genomes that are reliable for downstream analyses.

### ﻿CWDE and BSGC contents reveal lifestyles and pathogenicity potential of *Cytosporapiceae* EI-19(A) and *C.piceicola* EI-20

CWDEs are enzymes involved in carbohydrate synthesis or breakdown (Cantarel et al. 2009). They are enriched in many fungal species associated with plant hosts. Carbohydrate enzyme (CAZy) profiles of such fungi can provide clues to reveal their lifestyles and pathogenicity (Zhao et al. 2013). Functional modules of these enzymes are grouped into the classes: glycosyl transferases (GTs), glycoside hydrolases (GHs), polysaccharide lyases (PLs), carbohydrate esterases (CEs), enzymes for auxiliary activities (AAs), and carbohydrate-binding modules (CBMs) (Huang et al. 2018). The rich content of CWDE homologs in the genomes of hemibiotrophic and necrotrophic plant pathogens indicates their important role in cell wall breakdown. The reduced number of CAZy superfamilies (GHs, PLs, and CEs, in particular) can be attributed to biotrophy when organisms derive nutrients from living plant cells (O’Connell et al. 2012).

**Figure 6.**
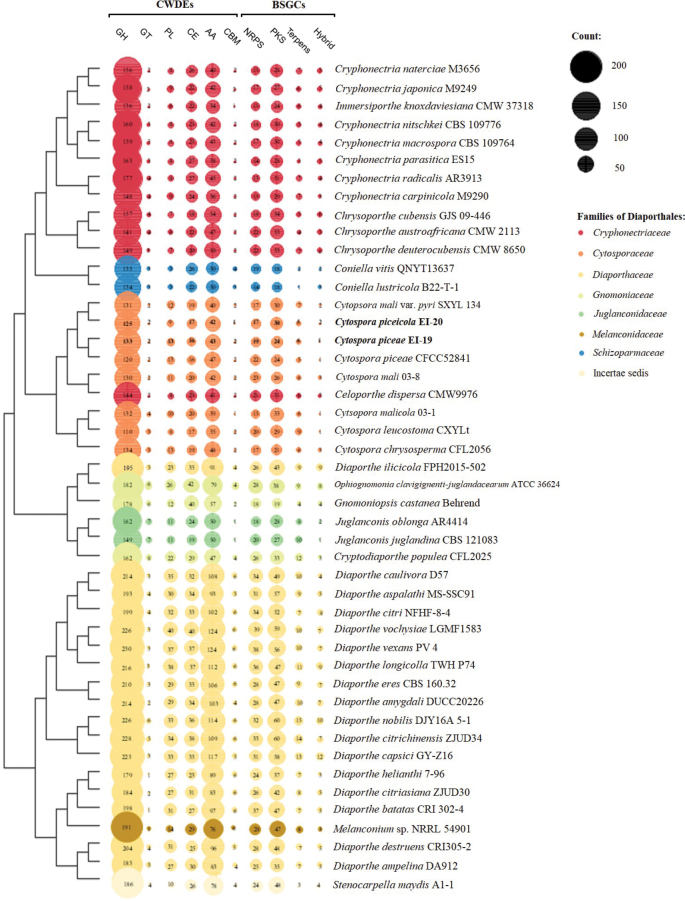
Contents of CWDEs and BSGCs predicted in genomes of Diaporthales species included in the analysis. The species are clustered based on the richness of each CWDE or BSGC group.

The number of secreted CWDEs in Diaporthales species included in the analysis ranged from 177 (*C.leucostoma* CXYLt) to 439 (*D.vochysiae* LGMF1583) (Fig. 6). A relatively low number of CAZy enzymes was identified in *C.piceicola* EI-20 (196 secreted out of a total of 498) and *C.piceae* EI-19(A) (209 secreted out of a total of 505). The strain of *C.piceae* CFCC 52841 contained a reduced set of GHs and a slightly larger number of AAs than *C.piceae* EI-19(A). The total number of CAZy detected in both studied species was slightly lower compared to those annotated for the model biotrophic fungus *Melampsoralarici-populina* (526) and hemibiotrophic species *Zymoseptoriatritici* (537) (Wang et al. 2022). *Colletotrichum* spp., known as hemibiotrophic plant pathogens mainly, also possessed larger arsenals of secreted CWDEs (from 246 (*Col.falcatum* Cf671) to 512 (*Col.fructicola* Cg38)) (Chen et al. 2022). *C.piceae* EI-19(A) and *C.piceicola* EI-20 were clustered with other *Cytospora* species and *Celoporthedispersa* CMW9976. This species of *Celoporthe* was found to be a non-pathogen or potential pathogen of Myrtales (Nakabonge et al. 2006) and carried 220 CAZy, while a highly virulent strain of *C.mali* 03-8 causing canker disease of *Malus* spp. (Wang et al. 2011) had a content of 207 such enzymes.

The genus *Diaporthe* had diverse sets of CWDEs across the species, from 325 for *D.helianthi* 7–96 to 439 for *D.vochysiae* LGMF1583. Interestingly, the former was well studied as a causal agent of sunflower stem canker (Baroncelli et al. 2016), but the latter was first isolated as an endophyte of *Vochysiadivergens* (Noriler et al. 2019). Based on the obtained evidence, it can be assumed that *C.piceae* EI-19(A) and *C.piceicola* EI-20 have a biotrophy-like relationship with the corresponding plant host. It can also be concluded that CWDE contents in both studied species allow for causing symptomatic canker disease that may unlikely lead to tree collapse.

Secondary metabolites (SM) consist of low-molecular-weight compounds that can play an important role in species pathogenesis. Nonribosomal peptides (NRP), polyketides (PKS), terpenes, and hybrid metabolites are synthesized by BSGC pathways (Keller et al. 2005). Produced chemical compounds (mycotoxins, in particular) are directly involved in fungus interactions with related hosts (Howlett 2006; Pusztahelyi et al. 2015).

The Diaporthaceae species (incl., *Melanconium* sp. and *Stenocarpellamaydis*) had the richest complement of SMs (from 77 (*D.ampelina* DA912) to 122 (*D.nobilis* DJY16A 5-1)) compared to Schizoparmaceae (from 36 (*Co.lustricola* B22-T-1) to 42 (*Co.vitis* QNYT13637)) (Fig. 6). Significant variability in the number of these gene clusters (PKS, especially) was previously noticed on the genus level for *Diaporthe* species (Hilário et al. 2022). Despite the relatively low number of BSGCs, *Coniella* species were found to be pathogens of some economically valuable crops (Çeliker et al. 2012; Zambounis et al. 2024). The *Cytospora* species shared similar proportions of different SMs: from 49 predicted for *C.chrysosperma* CFL2056 to 60 identified in *C.leucostoma* CXYLt. It is worth noting that the smallest content of CWDEs within the order was detected for the latter. There were 50 and 54 gene clusters involved in the secondary metabolism identified in *C.piceae* EI-19(A) and *C.piceicola* EI-20, respectively. *C.piceicola* EI-20 had a nearly identical repertoire of BSGCs as C.malivar.pyri SXYL 134, a variety of *C.mali* with reduced pathogenicity (Wang et al. 2011). Both species carried 30 polyketides, which were found to be involved in fungal virulence (Baker et al. 2006; Ruocco et al. 2018). That was notably higher than those predicted for *C.piceae* EI-19(A) (24). Generally, the *Cytospora* species possessed nearly the same number of SMs as many of the analyzed *Cryphonectria* species (e.g., 51 for *Cr.parasitica* ES15 and 55 for *Cr.carpinicola* M9290). The *Cryphonectria* species were previously related to hemibiotrophic or necrotrophic fungi and considered latent pathogens similar to some endophytes (Stauber et al. 2020). However, some were associated with important plant diseases such as chestnut blight (Rigling and Prospero 2018) and hornbeam decline (Cornejo et al. 2021). Functional genomics and transcriptomic studies have recently revealed an important role of NRPs in *C.mali*’s pathogenesis (Ma et al. 2016; Feng et al. 2020). This well-studied *Cytospora* species was previously classified as both necrotrophic (Yin et al. 2015) and hemibiotrophic (Stauber et al. 2020). 17 and 19 NRPs were predicted for *C.piceicola* EI-20 and *C.piceae* EI-19(A), respectively, smaller than those detected in *C.mali* 03-8 (23). However, the strain of *C.piceae* CFCC 5284 carried 22 of these SMs in its genome. Considering a reduced number of BSGCs identified in *C.piceae* EI-19(A) and *C.piceicola* EI-20, it is assumed that the lifestyle of both studied species can be multitrophic (closer to hemibiotrophic) with a capability to infect plant tissue and cause the CC disease quickly. The analysis also suggested that the different strains of *C.piceae* may have distinct pathogenicity and virulence characteristics.

### ﻿*Cytosporapiceae* EI-19(A) and *C.piceicola* EI-20 experienced gene family contraction during genome evolution

**Figure 7.**
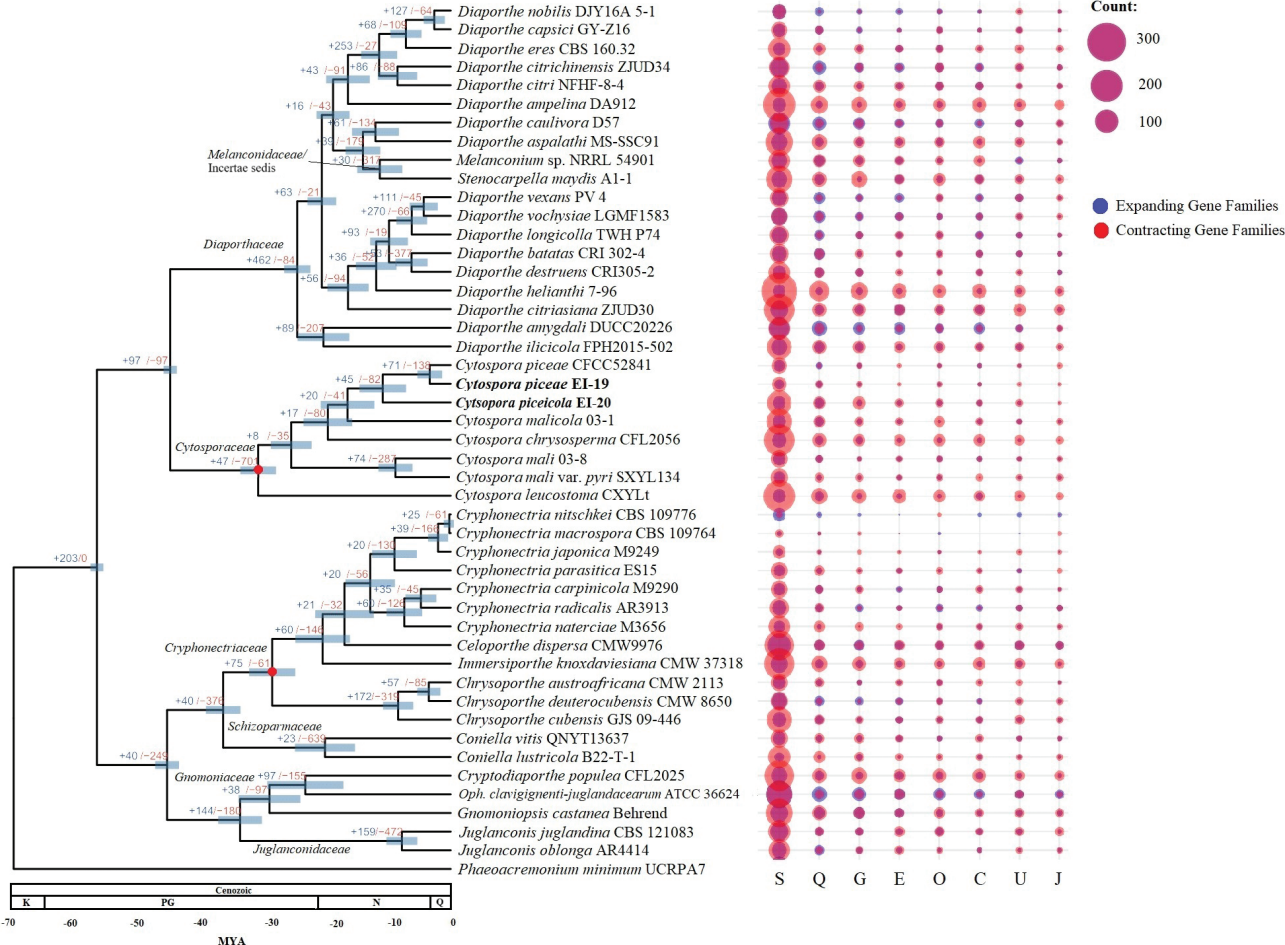
An ML tree showing phylogenomic relationships of *C.piceae* EI-19(A), *C.piceicola* EI-20, and other species of Diaporthales. Bootstrap support for all clades is 100. Blue and red numbers on nodes indicate the number of expanded and contracted gene families, respectively. Circles represent the number of expanded and contracted gene families for the top eight COG categories. Abbreviations: Q—Quaternary, N—Neogene, PG—Paleogene, K—Cretaceous, MYA—Million Years Ago.

A phylogenetic tree with estimated divergence times inferred based on the set of 1,706 SCOs is depicted in Fig. 7. The tree topology mainly supported the taxonomic positions of the families and genera within Diaporthales (Senanayake et al. 2017). The analysis showed the placement of *S.maydis* A1-1 within Diaporthaceae (Lamprecht et al. 2011). The strain of *Melanconium* sp. NRRL 54901 of Melanconidaceae (Castlebury et al. 2002) was also clustered with species of this family. The family-level classification (as Cytosporaceae) was confirmed for both *Cytospora* species analyzed in this study. The obtained divergence times matched those previously estimated for some Diaporthales families (e.g., 29.6 MYA for Cryphonectriaceae and 35.1 MYA for Cytosporaceae) (Guterres et al. 2018). The clade of *C.picea* and *C.piceicola* originated at 11-10 MYA, which might be considered a relatively late divergence compared to other Diaporthales such as *Co.vitis* QNYT13637 and *Co.lustricola* B22-T-1 or *D.amygdali* DUCC20226 and *D.ilicicola* FPH2015-502.

A total of 7,579 expanded and 13,984 contracted gene families were identified in the species of Diaporthales included in the analysis. The studied species harbored notably different numbers of expanded and contracted gene families (*C.piceae* (+46/-109), *C.piceicola* (+108/-319)), implying a moderate rate of gene contraction (+45/-82) (Fig. 7). For example, *C.leucostoma* CXYLt experienced a significantly higher rate of gene loss (+107/-638). In contrast, some species within the order have undergone gene family expansion (e.g., *D.amygdali* (+336/-250), *Oph. clavigignenti*-*juglandacearum* (+485/-369), *Cr.nitschikei* (+81/-16)). Gene family expansion is considered more beneficial for fungal pathogenesis. It may be linked to broader host adaptation, different reproductive strategies, and larger genomes carrying more pathogenicity-related genes (Ma et al. 2010). The species of Diaporthaceae harboring larger genomes compared to other Diaporthales experienced significant gene family expansion (+462/-84), while the reverse was true for the Cytosporaceae (+47/-701) and Schizoparmaceae (+23/-639) families. Host specificity and biotrophy characteristics were observed for the species after gene family contraction events (Zhang et al. 2018; Rogers et al. 2022). It was also found that the plant pathogenic species of Dothideomycetes contained more contracted gene families compared to saprophytes (Yu et al. 2022). This evidence can additionally support the biotrophic-like relationship of *C.piceae* EI-19(A) and *C.piceicola* EI-20 with *Picea* spp. specifically. On the other hand, gene family contraction may drive lifestyle differentiation and host shift in the studied species (Zhang et al. 2018).

The gene families with unknown function (S) were most frequently observed among the analyzed taxa of Diaporthales. Almost all the species experienced gene contraction of this category with some exceptions (e.g., *D.caulivora* D57 (+121/-95) or *Cr.nitschkei* CBS 109776 (+36/-7). The studied species showed moderate contraction of the S-categorized gene families (*C.piceae* EI-19(A) (+18/-53), *C.piceicola* EI-20 (+37/-154) than the other members of Cytosporaceae (*C.leucostoma* (+41/-269) or Diaporthaceae (*D.helianthi* 7–96 (+36/-349)). The secondary structure (Q) and carbohydrate metabolism and transport (G) gene families were also found to be contracted in *C.piceae* EI-19(A) and *C.piceicola* EI-20. But the latter underwent a notable loss (+7/-37) of the G category gene families compared to the former (+3/-8). A minor expansion (+9/-5) of the Q-categorized genes was observed for the strain *C.piceae* CFCC52841. This funding points out that the closely related species or different strains of the same species may differently affect corresponding hosts in terms of pathogenicity and virulence.

### ﻿*Cytosporapiceae* EI-19(A) and *C.piceicola* EI-20 constrain specific secretomes and effectomes within Diaporthales

Fungal plant pathogens secrete various proteins and metabolites to facilitate host infection. Among them are effectors secreted to reprogram host cells and modulate plant immunity (Stergiopoulos and de Wit 2009; Shao et al. 2021). The predicted secretome and effectome sizes varied significantly among 46 genomes of Diaporthales species included in the analysis. The number of secreted proteins ranged from 347 (*C.leucostoma* CXYLt) to 928 (*D.vochysiae* LGMF1583), or 3.45–5.55% of their respective proteomes (S/P). Effectome size ranged from 82 to 290, that is, 0.82–1.74% of the corresponding proteomes (E/P) (Table 3).

**Table 3.** Proteomes, secretomes, and effectomes of *C.piceae* EI-19(A), *C.piceicola* EI-20, and other Diaporthales species.

Family/Order	Strain	Accession number	Proteome	S/P (%)	E/P(%)
**Cryphonectriaceae**	*Celoporthedispersa* CMW9976	GCA_016584495	11,185	3.79	1.02
**Cryphonectriaceae**	*Chrysoportheaustroafricana* CMW 2113	GCA_001051155	12,161	3.6	0.96
**Cryphonectriaceae**	*Ch.cubensis* GJS 09-446	GCA_004802525	11,658	3.65	1.01
**Cryphonectriaceae**	*Ch.deuterocubensis* CMW 8650	GCA_001513825	12,430	3.61	1.03
**Cryphonectriaceae**	*Cryphonectriacarpinicola* M9290	GCA_014849695	10,827	4.1	1.22
**Cryphonectriaceae**	*Cr.japonica* M9249	GCA_014851275	10,290	4.19	1.24
**Cryphonectriaceae**	*Cr.macrospora* CBS 109764	GCA_004802535	10,300	4.24	1.24
**Cryphonectriaceae**	*Cr.naterciae* M3656	GCA_014850565	10,548	4.25	1.23
**Cryphonectriaceae**	*Cr.nitschkei* CBS 109776	GCA_004802565	10,761	4.05	1.16
**Cryphonectriaceae**	*Cr.parasitica* ES15	GCA_018104285	10,779	4.23	1.15
**Cryphonectriaceae**	*Cr.radicalis* AR3913	GCA_002179595	10,989	4.13	1.25
**Cryphonectriaceae**	*Immersiportheknoxdaviesiana* CMW 37318	GCA_021117315	10,442	3.88	1.15
**Cytosporaceae**	*Cytosporachrysosperma* CFL2056	NA*	10,304	3.52	0.96
**Cytosporaceae**	*C.leucostoma* CXYLt	GCA_003795295	10,045	3.45	0.82
**Cytosporaceae**	*C.mali* 03-8	GCA_000818155	10,564	3.73	0.98
**Cytosporaceae**	C.malivar.pyri SXYL134	GCA_000813385	10,248	3.78	1.16
**Cytosporaceae**	*C.malicola* 03-1	GCA_003795315	10,345	3.48	0.97
**Cytosporaceae**	*C.piceae* CFCC 52841	GCA_016508685	10,911	3.46	1.03
**Cytosporaceae**	***C.piceae* EI-19(A)**	**GCA_023375665**	**10,862**	**3.63**	**1.01**
**Cytosporaceae**	***C.piceicola* EI-20**	**GCA_023375675**	**10,742**	**3.68**	**1.02**
**Diaporthaceae**	*Diaportheampelina* DA912	GCA_001006365	13,155	4.9	1.55
**Diaporthaceae**	*D.amygdali* DUCC20226	GCA_021655905	14,520	5.39	1.74
**Diaporthaceae**	*D.aspalathi* MS-SSC91	GCA_001447215	14,023	4.98	1.32
**Diaporthaceae**	*D.batatas* CRI 302-4	GCF_019321695	14,366	5.18	1.5
**Diaporthaceae**	*D.capsici* GY-Z16	GCA_013364905	16,219	5.44	1.72
**Diaporthaceae**	*D.caulivora* D57	GCA_023703485	15,612	4.83	1.48
**Diaporthaceae**	*D.citri* NFHF-8-4	GCF_014595645	15,950	4.97	1.4
**Diaporthaceae**	*D.citriasiana* ZJUD30	GCA_014872975	14,345	5.07	1.67
**Diaporthaceae**	*D.citrichinensis* ZJUD34	GCA_014872995	16,322	5.48	1.73
**Diaporthaceae**	*D.destruens* CRI305-2	GCA_016859255	13,948	5.2	1.48
**Diaporthaceae**	*D.eres* CBS 160.32	GCA_024867555	15,503	5.14	1.64
**Diaporthaceae**	*D.helianthi* 7-96	GCA_001702395	12,718	4.8	1.31
**Diaporthaceae**	*D.ilicicola* FPH2015-502	GCA_023242295	14,231	4.66	1.36
**Diaporthaceae**	*D.longicolla* TWH P74	GCA_000800745	16,334	5.25	1.6
**Diaporthaceae**	*D.nobilis* DJY16A 5-1	GCA_023078575	16,460	5.42	1.68
**Diaporthaceae**	*D.vexans* PV 4	GCA_021188095	16,603	5.39	1.62
**Diaporthaceae**	*D.vochysiae* LGMF1583	NA*	17,434	5.32	1.66
**Gnomoniaceae**	*Cryptodiaporthepopulea* CFL2025	NA*	12,384	4.24	1.24
**Gnomoniaceae**	*Gnomoniopsiscastanea* Behrend	NA*	11,294	5.4	1.67
**Gnomoniaceae**	*Op. clavigignenti-juglandacearum* ATCC 36624	GCA_003671545	13,194	5.55	1.58
**Incertae sedis**	*Stenocarpellamaydis* A1-1	GCA_002270565	12,795	4.29	1.23
**Juglanconidaceae**	*Juglanconisjuglandina* CBS 121083	GCA_003012975	12,328	4.13	1.22
**Juglanconidaceae**	*J.oblonga* AR4414	GCA_003012965	12,012	4.37	1.22
**Melanconidaceae**	*Melanconium* sp. NRRL 54901	NA*	14,018	4.32	1.33
**Schizoparmaceae**	*Coniellalustricola* B22-T-1	GCA_003019895	9,148	3.95	0.94
**Schizoparmaceae**	*Co.vitis* QNYT13637	GCA_011317545	9,650	3.68	0.96
**Incertae sedis**	*Stenocarpellamaydis* A1-1	GCA_002270565	12,795	4.29	1.23

Notes: * The proteome was retrieved from MycoCosm [25].

**Figure 8.**
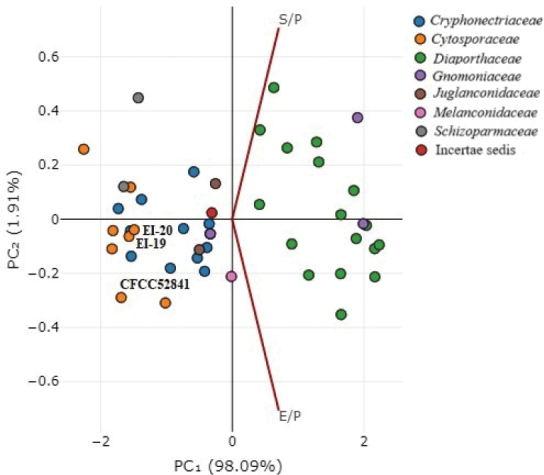
PCA plot showing secretome and effectome sizes of *C.piceae* EI-19(A), *C.piceicola* EI-20, and other Diaporthales species.

*C.piceae* EI-19(A) and *C.piceicola* EI-20 harbored very similar secretomes and effectomes, accounting for 3.63% (1.01%) and 3.68% (1.02%), respectively. It is worth noting that the strain *C.piceae* CFCC 52841 carried a smaller secretome (3.46%) with a higher number of effectors (1.03), which separated it from the studied strains (Fig. 8). Principal component analysis using the ratios of secreted proteins and effectors to respective proteomes grouped *C.piceae* EI-19(A) and *C.piceicola* EI-20 with the species of Cryphonectriaceae and Cytosporaceae. The closest species were *C.chrysosperma* CFL2056, *C.malicola* (*C.schulzeri*) 03-1, *Ch.austroafricana* CMW 2113, and *Ch.deuterocubensis* CMW 8650, the well-known canker-causing necrotrophic pathogens (Wang et al. 2011; Kepley et al. 2015; Dahali et al. 2023; Suzuki et al. 2023). Interestingly, *Melanconium* sp. NRRL 54901 and *S.maydis* A1-1 clustered with the Diaporthaceae species based on the contents of CWDEs and BSGCs had different secretome and effectome parameters.

A distinct separation of some Diaporthales taxa (Diaporthaceae, especially) can indicate genetic diversity variations of secreted proteins and effectors (Wang et al. 2022; Jia et al. 2023). This feature can be employed as a tool to reveal species lifestyles and pathogenicity potential of fungal species associated with plant diseases.

### ﻿*Cytosporapiceae* EI-19(A) and *C.piceicola* EI-20 harbor a wide range of virulence-related genes

Virulence genes encode proteins related to counteraction of host defense mechanisms that eventually lead to pathogen spread (Toth et al. 2003). The search of such genes was performed against the database of experimentally verified pathogenicity, virulence, and effector genes from fungal, oomycete, and bacterial pathogens of different hosts (PHI-base). The frequency of virulence-associated genes relative to their proteomes was nearly the same for the studied species (2.58–2.61%) (Table 4). The strain *C.piceae* CFCC 52841 had a relatively lower number of these genes (2.48%) in its proteome. The majority of the hits with PHI-base accessions for all three strains belonged to the categories with reduced virulence (144–148). The genes with unaffected pathogenicity (47–50) and loss of pathogenicity (32–35) were also defined in the analyzed species (Suppl. material 1). It showed that the main part of the identified genes (reduced virulence and loss of pathogenicity) was directly involved in fungal pathogenesis. The strains of *C.piceae* and *C.piceicola* EI-20 shared 238 virulence-associated genes. Notably, *C.piceicola* EI-20 carried 24 unique genes that was significantly more than *C.piceae* EI-19(A)(6) and *C.piceae* CFCC 52841(5). The findings additionally confirm that different strains or phylogenetically close species may have distinct pathogenicity and virulence characteristics. The content of virulence-related genes can be a strong indicator of fungus pathogenicity potential. For instance, the less pathogenic C.malivar.pyri SXYL134 (Wang et al. 2011) carried twice the lower number of these genes than the highly aggressive strain *C.mali* 03-8 (66 and 121, respectively) (Sun et al. 2023). This kind of analysis was also performed for other ascomycetous fungi associated with plant diseases such as *Diaporthe* (Hilário et al. 2022), *Neonectria* (Salgado-Salazar et al. 2021), etc. Because of different search parameters employed, the obtained results cannot be properly compared to those from the previous studies.

**Table 4.** Summary of predicted virulence-related genes after search against PHI-base.

Category	*C.piceae* EI-19(A)	*C.piceae* CFCC 52841	*C.piceicola* EI-20
Reduced Virulence	148	144	147
Unaffected Pathogenicity	50	47	50
Loss of Pathogenicity	32	32	35
Lethal	10	8	10
Increased Virulence	8	7	5
Other*	33	33	34
Total	281	271	281
Relative Frequency (%)	2.58	2.48	2.61

* Includes genes with different PHI-base annotations.

Host-induced gene silencing (HIGS) is a powerful alternative to traditional (e.g., chemical) treatments employed to protect plants from pathogenic organisms. The technology allows for the downregulation of the target genes in organisms associated with hosts that are not recalcitrant to genetic modifications (Hartmann et al. 2020; Koch and Wassenegger 2021). Target gene selection is the first and crucial step in HIGS. The pathogenicity and virulence factors identified in a pathogen isolated from a specific host significantly contribute to this process. For example, suppression of the effector genes (e.g., *AGLIP1*, *Avra*10) resulted in a reduction of fungus growth and disease development caused by *Rhizoctoniasolani* and *Blumeriahordei* in such economically important crops as barley, rice, and wheat (Nowara et al. 2010; Mei et al. 2024). Overall, properly selected candidate genes for HIGS enhance host resistance against a pathogen and help with disease control. The genes (effectors and virulence-associated genes, specifically) annotated for two *Cytospora* species associated with canker disease of spruce can be potential candidates for HIGS and other related gene engineering technologies.

## ﻿Conclusion

The study introduces genomes of *C.piceae* and *C.piceicola* sp. nov. assembled from Illumina reads. A number of pathogenicity-related factors, such as carbohydrate enzymes, secondary metabolites, effectors, and virulence-associated genes, were identified in the genomes of both studied species. The comparative genomics analysis revealed that *C.piceae* EI-19(A) and *C.piceicola* EI-20 are able to cause severe symptoms of canker disease in *Picea* spp. The findings contribute to understanding the biological processes that make these *Cytospora* species successful hemibiotrophic or biotrophic pathogens. However, more genomic studies should be conducted. For example, the transcriptomic approach using RNA-seq data can provide additional insights into the CC pathogenesis, showing the responses of a plant host during the early stage of infection and disease progression. Functional genomics techniques (e.g., gene knockout or RNA silencing) can be employed for phenotypic validation of genes with unknown functions that play an important role in fungal pathogenesis.

## Supplementary Material

XML Treatment for Cytospora piceae

XML Treatment for Cytospora piceicola

## ﻿Additional information

### Author ORCIDs

Evgeny Ilyukhin https://orcid.org/0000-0002-2358-0023

Yanpeng Chen https://orcid.org/0000-0002-2554-5272

Svetlana Markovskaja https://orcid.org/0000-0003-3111-6949

Ashwag Shami https://orcid.org/0000-0002-5336-038X

Sajeewa S. N. Maharachchikumbura https://orcid.org/0000-0001-9127-0783

### Data availability

This Whole Genome Shotgun project, including sequencing reads, has been deposited at DDBJ/ENA/GenBank under BioProject PRJNA796963, accession numbers JALQBB000000000 (*C.piceae* EI-19(A)) and JALQBC000000000 (*C.piceicola* EI-20).

## Supplementary materials

Supplementary material 1 PHI-base annotated genes identified in *C.piceae* CFCC52841 Data type xlsx https://binary.pensoft.net/file/1322564This dataset is made available under the Open Database License (http://opendatacommons.org/licenses/odbl/1.0/). The Open Database License (ODbL) is a license agreement intended to allow users to freely share, modify, and use this Dataset while maintaining this same freedom for others, provided that the original source and author(s) are credited.Evgeny Ilyukhin, Yanpeng Chen, Svetlana Markovskaja, Ashwag Shami, Sajeewa S. N. Maharachchikumbura
